# Novel versatile topologies and design optimization of wide-bandstop frequency selective surfaces for X-band, Ku-band and millimeter-wave applications

**DOI:** 10.1038/s41598-023-28922-4

**Published:** 2023-02-02

**Authors:** Rao Shahid Aziz, Slawomir Koziel, Leifur Leifsson, Stanislaw Szczepanski

**Affiliations:** 1 Department of Engineering, Reykjavik University, Reykjavik, Iceland; 2 Faculty of Electronics, Telecommunications and Informatics, Gdansk University of Technology, Gdansk, Poland; 3 School of Aeronautics and Astronautics, Purdue State University, West Lafayette, IN USA

**Keywords:** Engineering, Physics

## Abstract

Novel designs of frequency selective surface (FSS) are presented for wideband applications in X, Ku and mmWave (millimeter Wave) bands. Two identical metallic layers of FSS are imprinted on both sides of the RO4003 substrate. The geometry parameters are optimized to maximize the bandstop at the specified in-band maximum transmission level of −10 dB; satisfaction of the latter condition is enforced through appropriate formulation and handling of the design constraints. The proposed structure is versatile and can be readily re-designed for various operating bands. For the sake of illustration, two instances of the FSS were developed. Design 1 exhibits broad bandstop of 9.8 GHz at the X- and Ku-bands, whereas the bandstop of Design 2 is 33.5 GHz at the mmWave band. The two FSS unit cell designs share the same base topology, but specific dimensions are adjusted to operate within the lower and the higher bands, respectively. The unit cell is symmetrical, therefore, ensures an excellent resonance stability performance with respect to different polarizations (TE and TM) and incidence angles. For proof of concept only FSS Design 1 is fabricated and measured in an anechoic chamber. The simulated and measured results exhibit good agreement. Extensive benchmarking against state-of-the-art FSS designs from the literature corroborates the advantages of the proposed topology in terms of design novelty, topological versatility, compact size, and wide bandstop response as compared to the previously available designs.

## Introduction

Frequency selective surfaces (FSSs) are arrays of periodic elements, referred to as unit cells. These can be 2D or 3D structures consisting of metallic layers on dielectric substrates^1–3^. FSS realizes either transmission or reflection characteristics, depending on the unit cell design. FSSs have been investigated for decades and found applications in antenna reflectors, absorbers, polarizers, hybrid radomes, spatial filters, etc. Nowadays, broad-bandstop FSSs are widely used in electromagnetic interface (EMI) shielding^4–8^, reduction of mutual coupling in multiple-input-multiple-output (MIMO) antennas^9–14^, as well as for enhancing antenna directivity^15–21^.

The aforementioned variety of applications led to the development of an equal variety of different FSS structures, reported and analyzed in the literature. These include metamaterials, electromagnetic (EM) bandgap designs, and FSSs. Notwithstanding, FSS components are more suitable in terms of the fabrication cost, ease of manufacturing, and low profile. In^6^, a 2.5D periodic layer of via-based FSS structure has been proposed for shielding applications in mmWave. However, the proposed FSS structure is complex for fabrication and bulky. Another single-layer FSS for wideband shielding was reported in^7^. It features 7.5 GHz stopband to provide shielding in both X- and Ka-bands from 6.5 to 14 GHz range. Although many other wideband FSS designs can be found in the literature^22–27^, most of them lack stability in terms of polarization and the incidence angle, whereas others are bulky and complicated to manufacture.

One of the recent applications of FSS is a reduction of mutual coupling between closely coupled antenna array elements. Coupling reduction can be realized using several distinct techniques^28^. One of possible options is to employ low index metamaterial loaded in front of the end-fire antennas^29^. Other possibilities include cutting a slot in the common ground plane between elements to reduce the surface currents^30–34^, or introduction of a neutralization line between the two coupled elements^35–38^. On the other hand, FSS and EM bandgap have attracted considerable attention due to their promising performance in terms of wideband operation and radiation pattern preservation in the microwave^39–41^ and mmWave areas^9,42,43^.

Yet another application of wideband FSS is in highly directive antennas, where FSS are used as a superstrate layer^44–46^. In^47^, a square loop shape FSS is utilized as a superstrate to enhance the directivity of aperture-coupled microstrip antenna (ACMA). Apart from the enhancement of directivity, the FSS incorporated as a superstrate layer (either as a single element of in combinations), can also be applied for improving the bandwidth^27^, as well as controlling polarization^23^ and radiation pattern^44,48^. In^49^, an Ultra-thin wideband metasurface polarization converter is presented. The proposed structure is used for linear conversion i.e., X polarized to Y polarized and vice versa. The wide bandwidth is 10.81 GHz (10.57–21.38 GHz) above 89%. In^50^, compact double square shaped design of a Metamaterial Absorber (MA) is investigated. It has wideband absorption ranges from 14.44 to 27.87 GHz. Thus, a bandwidth of 13.43 GHz at − 10 dB. It covers Ku (12–18 GHz) and K (18–27 GHz) bands. Also, in^51^, another compact wideband metamaterial absorber for Ku band applications is proposed. It covers 11.39 to 20.15 GHz with a bandwidth of 8.76 GHz which fully covers the Ku band.

The literature offers numerous FSS designs^1–60^. Notwithstanding, in the context of many application areas, some of the FSS properties still need considerable improvement, one of which is the broad frequency range of operation. Achieving this does not only require appropriate selection of the structure topology but also meticulous adjustment of geometry parameters, which is an intricate task by itself.

This communication proposes novel structures of single-layer wide bandstop FSSs. The FSS consists of two geometrically identical metallic layers separated by the Rogers RO4003 substrate. The design is compact, gives stable polarization and the incidence angle response at different angles. Furthermore, it is versatile and can be tuned to both lower and higher operational frequency bands. To demonstrate this feature, two specific FSS structures are designed. Design 1 operates within the X- and Ku-bands, whereas Design 2 operates in the mmWave band. Design 1 exhibits a broad −10 dB bandwidth of 12.16 GHz (from 6.81 to 18.97 GHz), which is equivalent to about 94.5% fractional bandwidth with respect to the center frequency (12.4 GHz). FSS Design 2 features a −10 dB bandwidth of 33.5 GHz (from 22.28 to 55.78 GHz, equivalent to 85.8% fractional bandwidth with respect to the center frequency of 39 GHz). One of the critical components of the design process is simultaneous optimization of all unit cell dimensions. In the course of this process, the two objectives (bandwidth enhancement and preservation of the in-band transmission level) are aggregated into a scalar cost function using a penalty function approach. The unit cell is symmetric, therefore, stable TE/TM modes and angle of incidence are obtained. A panel of 50 × 50 unit cells of FSS Design 1 operates within the X- and Ku-bands has been fabricated and experimentally validated. Design 2 which operated in mmWave band has analyzed in CST microwave solver. Comprehensive benchmarking indicates that the proposed design has a competitive edge over the solutions reported in the literature, especially in terms of the operating bandwidth and the size.

The originality and the technical contribution of this work can be summarized as follows: (i) the development of a novel topology of the FSS unit cell that is readily scalable to different operating bandwidths (here, demonstrated for X-/Ku-band and mmWave band), (ii) demonstrated competitive performance of the proposed FSS in terms of the achievable bandstop (close to 100% for both specific designs), and (iii) the development of the customized simulation-based optimization technique that explicitly targets bandwidth improvement while offering a precise control over the in-band transmission level.

## Methods

### FSS unit cell design

This section describes the geometry of the proposed FSS unit cell along with the qualitative analysis of its properties and the optimization procedure. The two specific designs rendered for the operation in the X-/Ku-bands (Design 1), and mmWave band (Design 2) will be discussed in section “Conclusion”.

### Unit cell geometry

Wide-bandwidth FSS is an ideal candidate for a variety of applications, as elaborated on in section “Introduction”. In order to obtain this feature, one of the preferred techniques was to exploit multiple resonances implemented using dual metallic layers^26^. Based on a similar approach, a single dielectric layer FSS is proposed in this work. It is composed of two identical metallic layers imprinted on both sides of the dielectric substrate. The topology consists of metallic strips arranged in a cross pattern, enclosed in a circle printed on the dielectric material. This architecture results in a wide bandstop transmission response as demonstrated later in the paper. Furthermore, it ensures compact design with an added possibility to readily tune the structure to different operating frequencies by adjusting the inner and the outer radii of the circle, and the width of the strips.

**Figure 1:**
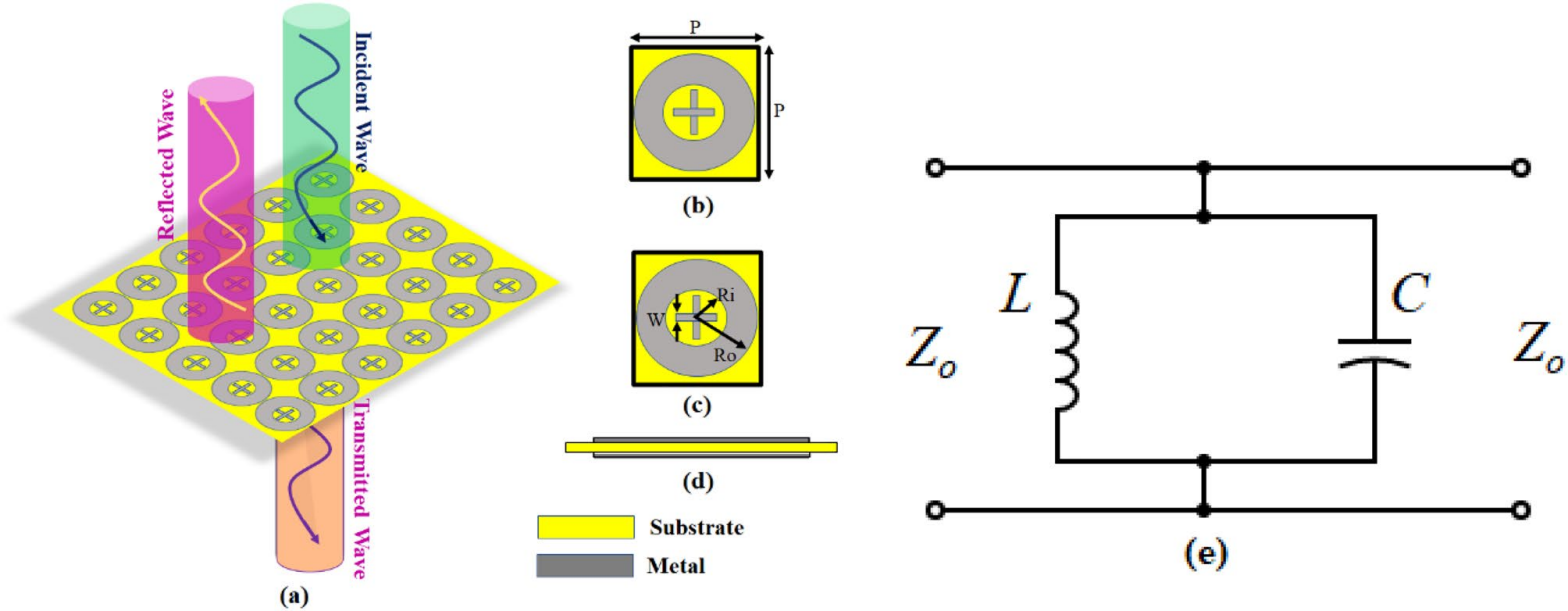
Proposed FSS unit cell geometry, (**a**) incidence of EM wave on FSS Panel, (**b**) top view, (**c**) bottom view, (**d**) side view, (**e**) equivalent circuit model.

The FSS is designed on Rogers RO4003C (lossy) dielectric material with material thickness, t = 0.508 mm, relative permittivity, ε_r_ = 3.38, and the dissipation factor tanδ = 0.0027^61^. The details of the geometry configuration of the proposed FSS are shown in Fig. 1. The variables parameterizing the unit cell are labeled in Fig. 1. These are P, R_o_, R_i_, L, and W (all dimensions in mm). The FSS unit cell is characterized using the frequency-domain solver of CS.

### Basic properties and parametric studies

**Figure 2:**
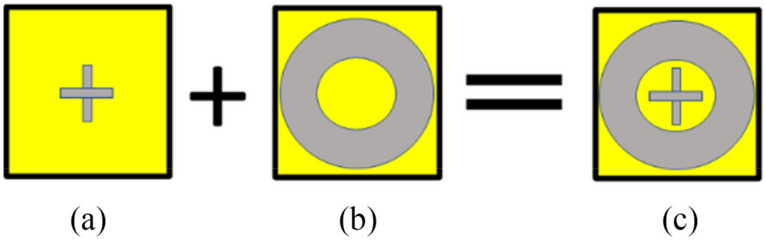
Generation of the proposed FSS geometry, (**a**) cross-dipoles, (**b**) outer ring, and (**c**) a combination of cross-dipoles and ring.

**Figure 3:**
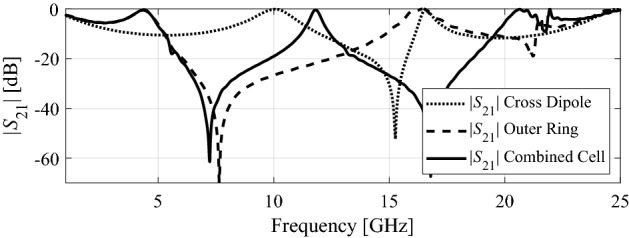
Comparison of transmission coefficients (|S_21_|) of the cross-dipoles, the outer ring, and their combination.

This section explains a formation of the proposed wideband FSS. Figure 2 shows the FSS geometry, which consists of two well-known shapes, i.e., two crossed dipoles and an outer ring^7,18^ printed on both sides of the FSS unit cell. The transmission coefficient corresponding to the two cross dipoles exhibits a stopband from 11.7 to 16.8 GHz below −10 dB, as indicated in Fig. 3. CST Microwave Studio simulator, using the periodic boundary conditions^62^.

Likewise, an outer ring yields a stopband transmission from 5.3 to 14.8 GHz (again, at the –10 dB level). When both the shapes are combined, two broad stopbands are obtained in the range from 5.3 to 10.7 GHz and from 12.1 to 20.4 GHz below −10 dB, as also shown in Fig. 3. A passband of 1.4 GHz can be observed in the combined structure transmission coefficient, from 10.7 to 12.1 GHz. The stopband of the proposed FSS can further enhanced through a parametric study of the combined unit cell parameters (R_i_ and L). A procedure for rigorous optimization of the unit cell will be explained in the next subsection.

**Figure 4:**
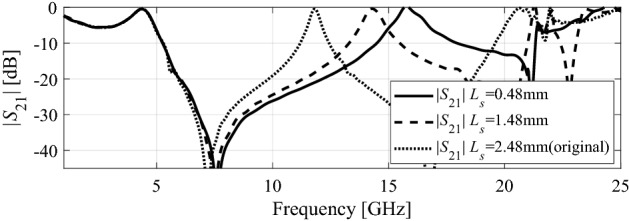
Comparison of transmission coefficients (|S_21_|) of the FSS unit cell for different lengths of the cross-dipoles.

A parametric study is carried out to improve the proposed FSS design. Two parameters L_s_ and R_i_ are analyzed in the context of bandwidth enhancement and the resonance behavior. Figure 4 demonstrates the dependence of the transmission coefficient on the cross dipole length. The objective is to obtain a possibly broad stopband FSS. The length of each cross dipole *L*_s_ is decreased from 2.48 mm (original) to 0.48 mm. It can be seen that when the length Ls is decreased to 0.48 mm, the second transmission zero, *f*_z2_ (originally allocated at 16.8 GHz) is shifted to a higher frequency of 21 GHz, whereas the first transmission zero, f_z1_ is slightly shifted from 7.22 GHz to 7.6 GHz. Likewise, the second transmission pole, *f*_p2_ (originally allocated at 11.8 GHz) is shifted to higher frequency of 15.7 GHz, and the first transmission pole *f*_p2_ remains at 4.4 GHz. Based on this parametric study, the FSS bandwidth is increased by about 3.35 GHz.

**Figure 5:**
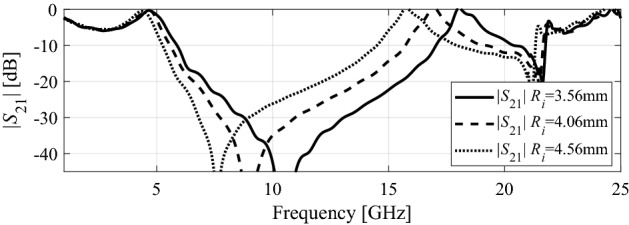
Comparison of transmission coefficients (|S_21_|) of the FSS unit cell for different inner radii of the outer ring.

Subsequently, the effect of the ring radius of the FSS unit cell is studied. The inner radius of the ring R_i_ is decreased from 4.56 to 3.56 mm while keeping the length of the cross-diploes equal to 0.48 mm. Figure 5 shows the effect of altering *R*_i_. When it is decreased to 3.56 mm, the second transmission zero, *f*_z2_ is shifted from 21 GHz to a higher frequency of 21.6 GHz. At the same time, the first transmission zero, *f*_z1_ is shifted from 7.6 to 10.7 GHz. Similarly, the second transmission pole, f_p2_ is shifted to a higher frequency, i.e., from 15.7 to 18 GHz. The first transmission pole *f*_p2_ is shifted from 4.4 to 4.7 GHz. Consequently, the −10 dB bandwidth is increased by 2.54 GHz.

The Fig. 1e shows the equivalent circuit model of the proposed FSS structure^56–60^. The unit cell contains an inductor and capacitor, which forms a parallel LC circuit. The LC components are connected via parallel transmission lines with a characteristic impedance of free space, Z_o_ = 377 ohms. This combination forms a bandpass filter with center frequency *f*_*o*_, which can be approximated by Eq. (1).

1 $$f_{o} = \frac{1}{{2\pi \sqrt {LC} }}$$

Using parametric study, the proposed design can be utilized as a wideband stop filter. Further tuning of the geometry parameters is carried out using a rigorous numerical optimization procedure described in section “Unit cell geometry”.

### Design optimization procedure

The approximate adjustment of the operating bandwidth of the proposed FSS unit cell can be achieved by scaling the outer size *P* along with the inner/outer ring radii. However, ensuring maximum possible bandwidth as well as maintaining the required maximum transmission level (here, −10 dB) within that bandwidth is a matter of meticulous tuning of all FSS parameters. This involves several components, in particular, an appropriate parameterization of the cell, formulation of the objective function, and the choice of the optimization algorithm.

For the purpose of design optimization, the unit cell is parameterized in a relative manner to ensure geometrical consistency of the structure, in particular, to avoid overlapping the parts (e.g., the strips and the ring) as well as keeping the metallization parts within the substrate outline. The following vector of optimizable variables is employed: *x* = [P R_or_ R_ir_ L_r_ W]^T^, with the original parameters related to the components of the vector x as follows: R_o_ = R_or_P, R_i_ = R_ir_R_o_, L = L_r_R_i_, and a single constraint of the form L^2^ + W^2^/4 ≤ R_i_^2^. The constraint is to ensure that the stripes are entirely within the inner circle. The parameter space is defined as P_min_ ≤ P ≤ P_max_, 0 < R_or_, R_ir_, L_r_ < 1, and W_min_ ≤ W ≤ W_max_. Note that using relative parameters allows for simplifying the parameter space geometry (only one nonlinear constraint is used), which facilitates cell optimization from numerical standpoint.

**Figure 6:**
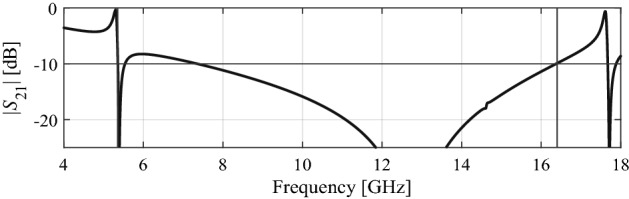
Definition of the FSS unit cell bandstop: *f*_L_ and *f*_H_ denote the lower and upper ends of the bandstop, whereas S_max_ stands for the maximum transmission level within the operating bandwidth.

The design objective is to maximize the −10 dB transmission (bandstop) bandwidth, which is defined as B = f_H_ – f_L_, with f_L_ and f_H_ being the frequencies defining the lower and upper ends of the bandstop (cf. Figure 6), while ensuring that the transmission (here, denoted as |S_21_|) does not exceed −10 dB therein. By denoting the maximum in-band transmission as S_max_, the objective function to be minimized is defined as

2 $$U\left( {\varvec{x}} \right) = - \left[ {f_{H} \left( {\varvec{x}} \right) - f_{L} \left( {\varvec{x}} \right)} \right] + \beta \left[ {(S_{max} []^{2} } \right]$$

Note that the primary objective is negative bandwidth (to turn the problem into a minimization task), whereas the constraint on the maximum transmission level violation is handled implicitly using the penalty term. This formulation allows for avoiding objective function discontinuities that would take place if—during the optimization process—violations of the –10 dB threshold occur within the operating band.

The optimization problem is then posed as

3 $${\mathbf{x}}^{*} = \arg \min \{ {\mathbf{x}}:U({\mathbf{x}})\}$$

and solved using the trust-region gradient search^63^ with numerical derivatives. The algorithm is implemented in Matlab, and the computational model of the FSS cell is evaluated using the custom Matlab-CST socket permitting batch-model simulations of the structure.

### Simulation results and discussion

This section provides an in-depth analysis of the properties of the two specific designs of the proposed FSS, optimized for X/Ku band and for mmWave band, respectively. These two designs are discussed to elaborate on the topological versatility of the proposed FSS. The analysis is carried out in terms of their corresponding current distributions at different operating frequencies as well as angle of incidence for the TE and TM modes.

### Design 1: X-/Ku-band

**Figure 7:**
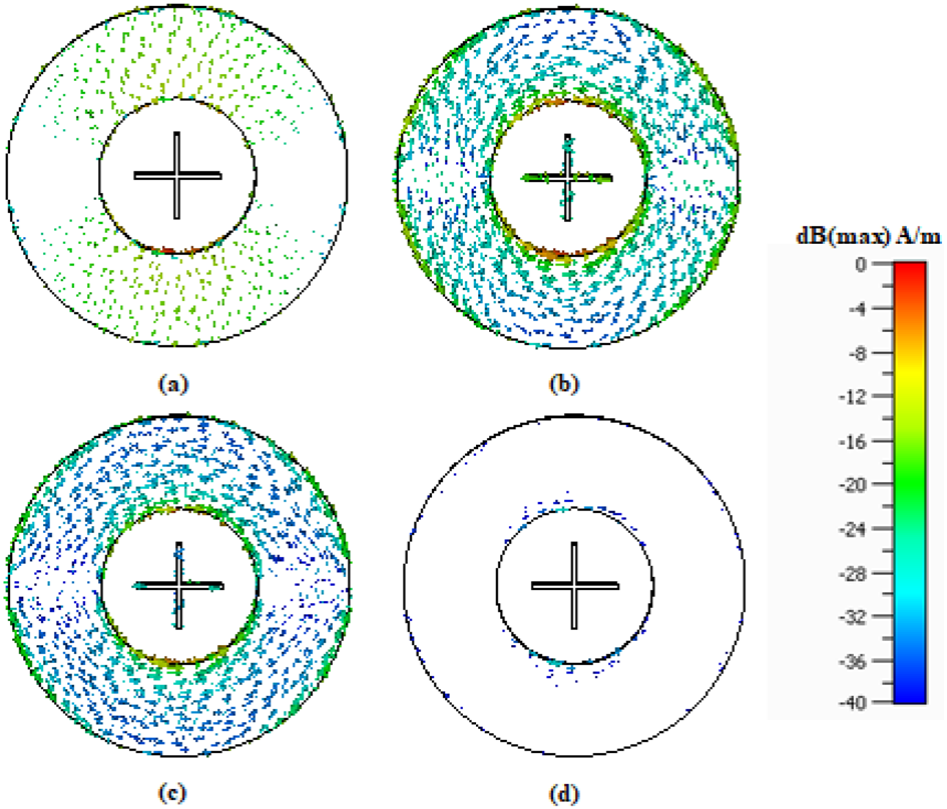
Surface current distribution on the FSS Design 1 at (**a**) 5 GHz, (**b**) 10 GHz, (**c**) 15 GHz, (**d**) 20 GHz.

The first of the two considered design is the FSS unit cell optimized for X (8–12 GHz) and Ku (12–18 GHz) bands. The objective is to ensure wide stopband at both X and Ku bands simultaneously. The total size of the unit cell Design 1 is 11.53 × 11.53 mm^2^. The combination of the two cross-dipoles and the outer ring on both sides of the substrate allow it to resonate at the desired bands. Additionally, the FSS dimensions are optimized using the methodology of section “Unit cell geometry” to maximize the bandwidth, which leads to operating frequency band is from 6.81 to 18.97 GHz (at the −10 dB level), which provides a sufficient margin for both the X and the Ku band. The reflection coefficient remains 0 dB over these frequency range. This design is further studied in order to obtain a better insight into the physics of the unit cell. Also, a panel of size 263 × 263 mm^2^ has been experimentally validated in the next section. Figure 7 shows the surface current distribution on the proposed FSS at different frequencies. A uniform plane wave is incident in the + z direction and the electric field is induced on the surface of the unit cell. It can be observed that current density assumes smaller values at the higher and lower frequencies that are out of the stopband, see Fig. 7a, d, whereas in the operating frequency band (6.81–18.97 GHz), the current density is increased, cf. Fig. 7b, c.

**Figure 8:**
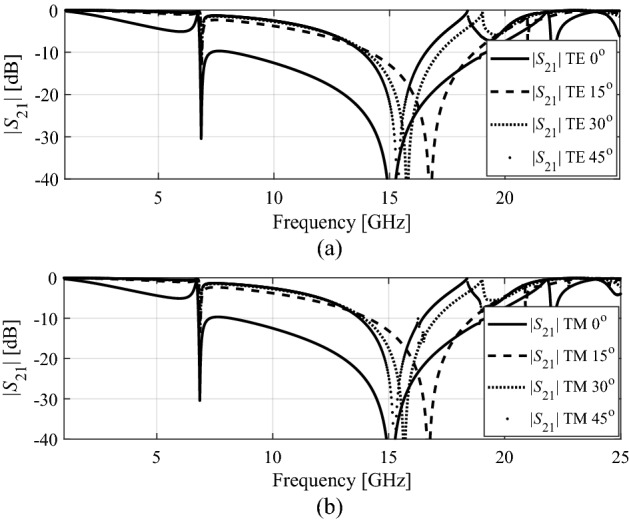
Design 1: (**a**) transmission coefficients for TE mode, (**b**) transmission coefficients for TM mode at different incidence angles.

Figure 8 shows the transmission coefficients for TE and TM modes at different angles of incidence, varying from 0 to 45 degrees. The plots indicate that both the TE and TM polarizations the transmission bandwidth is reduced essentially with respect to the incidence angle. It can be noticed that the unit cell geometry is symmetric therefore, transmission coefficients for TE and TM modes obtained same resonance response.

### Design 2: mmwave band

**Figure 9:**
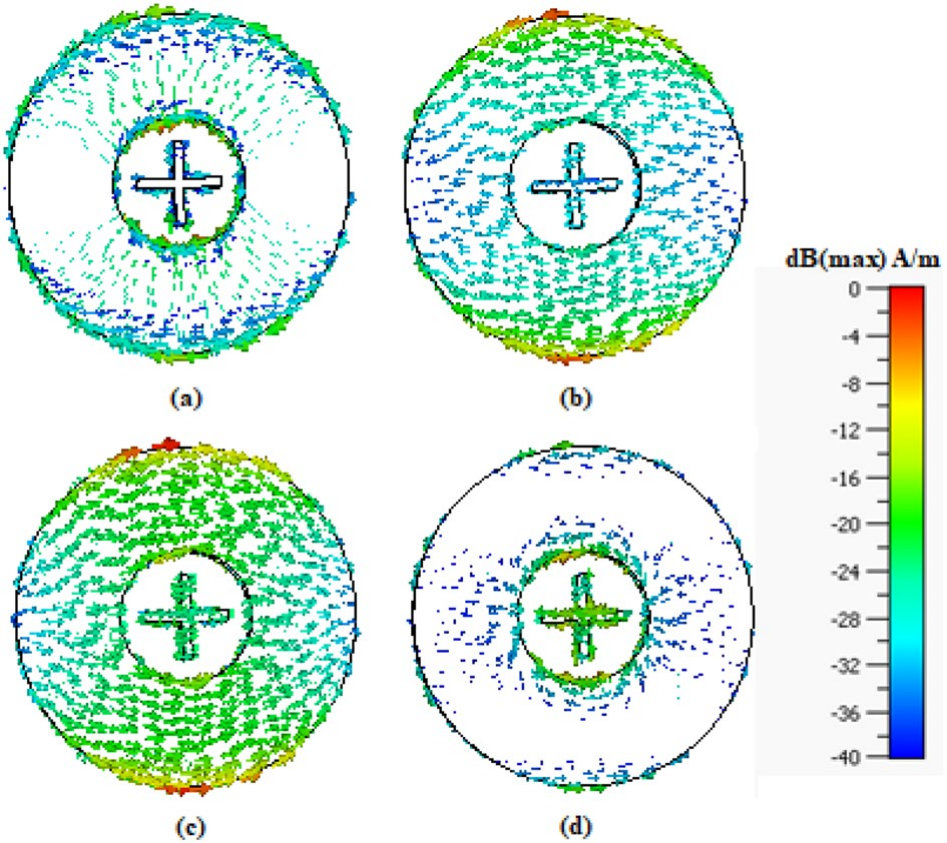
Surface current distribution on the FSS Design 2 at (**a**) 17 GHz, (**b**) 25 GHz, (**c**) 40 GHz, and (**d**) 58 GHz.

The proposed FSS geometry is versatile and can be re-designed to operate at higher frequencies. For the sake of illustration, Design 1 of the FSS is scaled down and re-optimized for mmWave operation. The resonating components of Design 2 are same two cross-dipoles and an outer ring printed on both sides of the substrate. Now, the total size of the unit cell Design 2 is 4.24 × 4.24 mm^2^. Combination of these components gives a wide range of stopband at higher frequencies. The achieved stopband at mmWave band is as broad as from 22.28 to 55.78 GHz. The reflection coefficient remains 0 dB over these frequency range. The suitable panel for mmWave band is 103 × 103mm^2^ which can also be experimentally validated. However, in this work only Design 1 is fabricated. The surface current distribution has been shown in Fig. 9. Similarly, as for Design 2, the current density is diminished for the frequencies which are out of stopband on both the dipoles and the outer ring (Fig. 9a, d). On the other hand, the current density is higher at the frequencies being inside the stopband (Fig. 9b, c).

**Figure 10:**
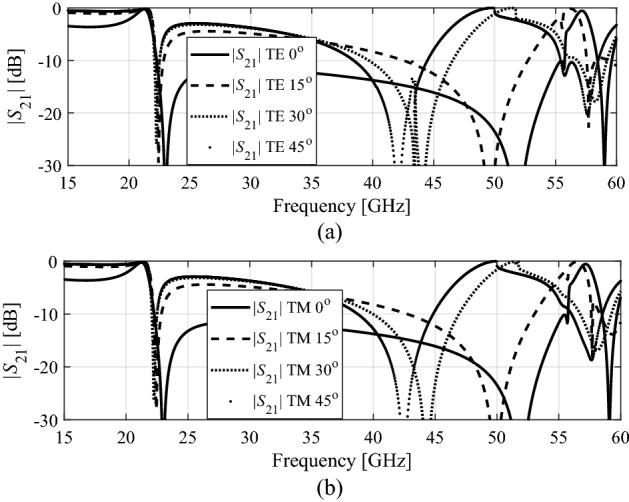
Design 2: (**a**) transmission coefficients for TE mode, (**b**) transmission coefficient for TM mode at different incidence angles.

Design 2 is also studied in Fig. 8 in terms of the angle of incidence. Figure 10 shows the transmission coefficients for TE and TM modes at different angle, varying from 0 to 45 degrees. The TE and TM polarizations show bandwidth reduction by increasing the oblique incidence angle, which was to be expected as both designs share the same fundamental topology. Also, in Design 2 it can be noticed that the unit cell geometry is symmetric therefore, transmission coefficients for TE and TM modes obtained are identical.

### Experimental validation

This section discusses the measurement and simulation results of the realized FSS panel, populated with the copies of the proposed unit cells. Experimental validation includes investigating the properties of the FSS under normal and other incidence angles.

### Normal incidence and polarization stability

**Figure 11:**
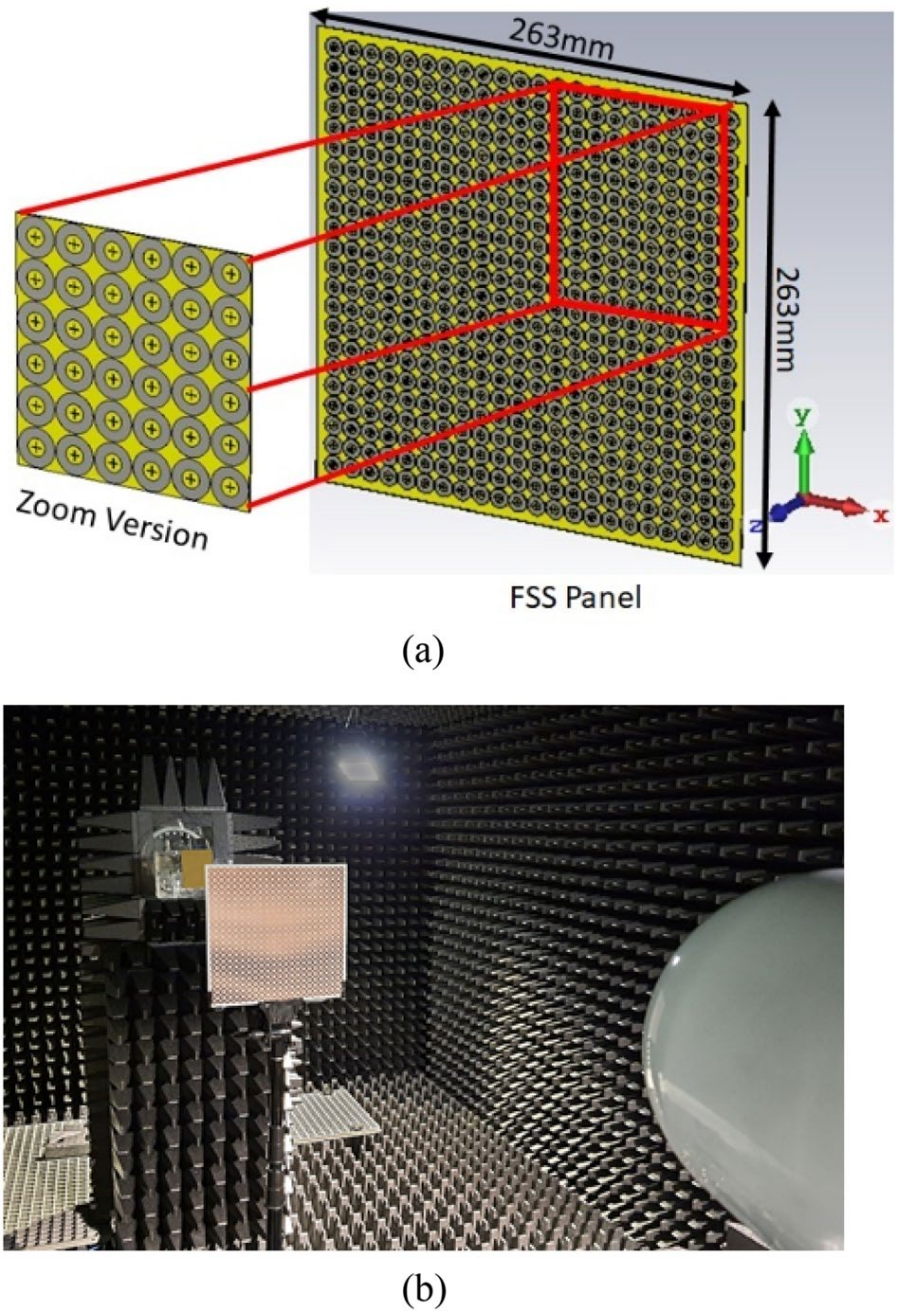
FSS panel of Design 1, (**a**) CST simulated environment and (**b**) measurement setup.

A prototype FSS of Design 1 is fabricated on Rogers RO4003C (lossy) panel of the size 263 × 263 mm^2^. The FSS panel consists of 22 × 22 unit cells in the xy-plane. The transmission magnitude using two reference antennas with the operating bandwidth 2 to 18 GHz. The measurement setup with FSS panel under test is shown in Fig. 11. The reflection coefficient remains at 0 dB from 6.81 to 18.97 GHz. The reference horn Antenna 1 features 3-dB beamwidth of 45° in the E-plane and 20° in the H-plane, whereas the horn Antenna 2 exhibits 3-dB beamwidth of 120° in both the E- and H-planes. In the measurement process the incidence angles from 0° to 45° are considered. The network analyzer, Agilent technologies PNA-X N5242A with 10 MHz to 40 GHz frequency range is used.

**Figure 12:**
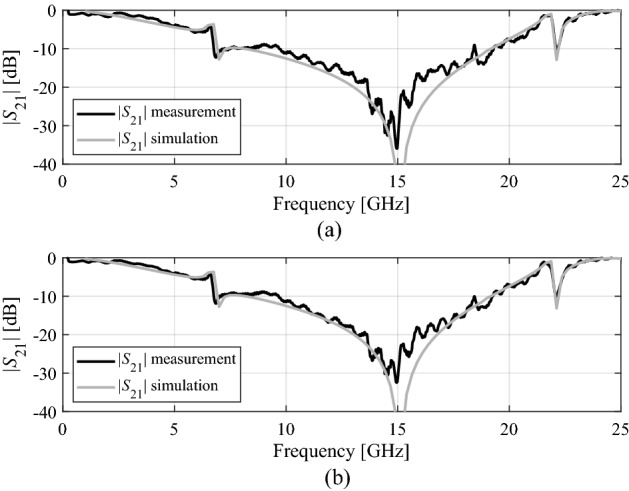
Measured and simulated results, (**a**) TE mode, (**b**) TM mode for normal incidence.

The transmission characteristics without the FSS panel are measured as well for the sake of calibration. The comparison between the simulated and the measured data for both TE and TM polarization for the incident angle of zero degrees is shown in Fig. 12. It can be observed that the agreement between simulated and measured results is reasonable. The slight variations are due to the reflections and scattering of the EM waves during measurements.

### Oblique incidence and polarization stability

**Figure 13:**
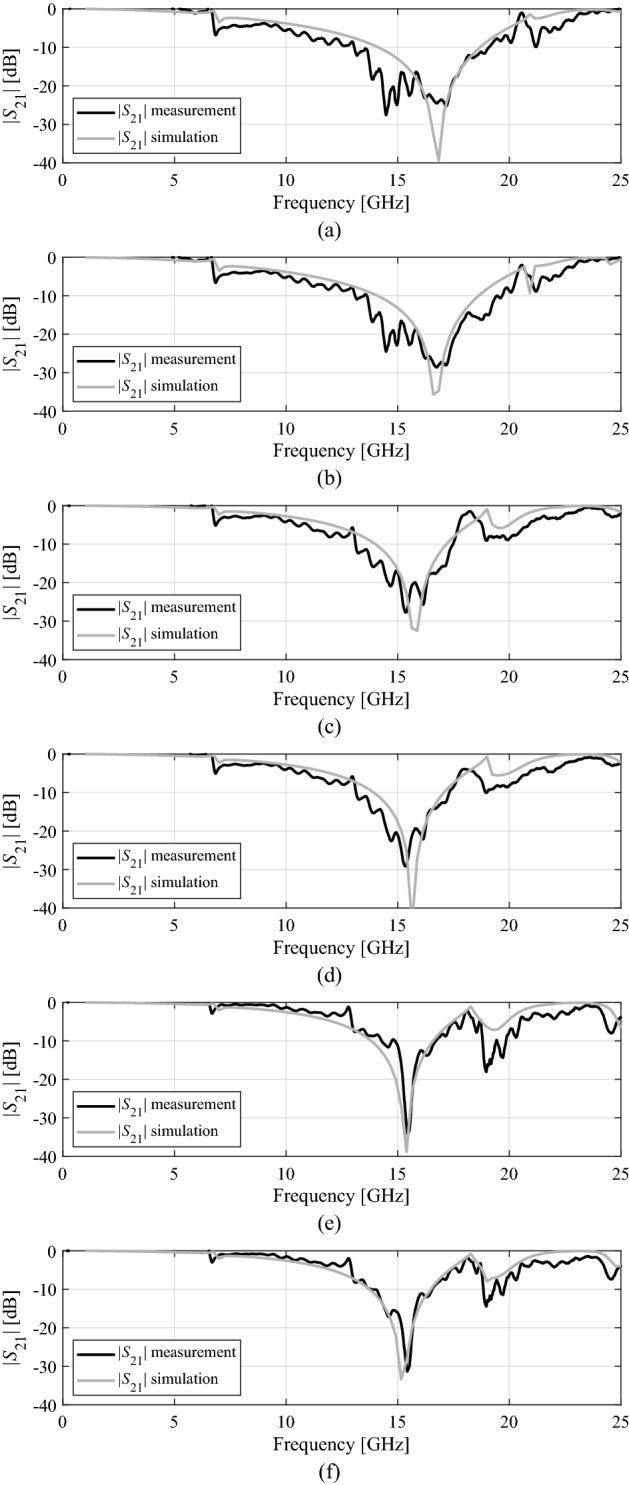
Measured and simulated results, (**a**) TE mode at 15°, (**b**) TM mode at 15°, (**c**) TE mode at 30°, (**d**) TM mode at 30°, (**e**) TE mode at 45°, (**f**) TM mode at 45°.

To investigate the oblique incidence performance, a Floquet port analysis has been performed in simulation. The measurement of the oblique incidence and polarization stability is carried out in the same setup by manually rotating the FSS panel of Design 1 to the desired angles. Figure 13 shows the angle transmission magnitudes for the TE and TM polarized waves up to an angle of 45° measured from the normal to the oblique. It can be seen that the bandwidth of FSS panel is reduced in the oblique incidence but stable TE and TM polarization is obtained for all of the incident angles.

### Benchmarking

FSS is a well-known technique however, the design methodology implemented in this work is novel along with the specific geometry of the unit cells. Table 1 shows the performance comparison of the proposed FSS design versus state-of-the-art designs reported in the literature. Both combined with the customized optimization procedure leads to superior performance as elaborated on in Table 1. The comparison is conducted in terms of the fractional bandwidth, bandstop frequency range, number of FSS unit cell metallic layers, unit cell size (mm^2^), substrate material and radiating Part of the unit cell. It can be observed the proposed FSS geometry enables competitive fractional bandwidth while maintaining a very low profile as compared to other reported designs. The proposed FSS geometry is versatile and readily scalable for higher frequency.

**Table 1:** Proposed FSS design versus state-of-the-art literature solutions.

Design	Bandwidth (%)	Bandstop bandwidth (GHz)	Number of metallic layers	Unit cell size (mm^2^)	Substrate	Radiating part
^7^	73.2	6.5–14	2	12 × 12	FR4	Cross-dipole and outer ring
^22^	51.1	5–16	2	15 × 15	FR4	Three cascaded layer copper strips
^23^	110.0	3.05–10.65	4	18 × 20	FR4	Square loop
^24^	31.6	8–11	2	Not Available	FR4	Cross dipole
^25^	54.5	4–7	2	10.6 × 10.6	FR4	Square loop
^26^	85.5	8–20	2	6.8 × 6.8	F4B-2	Square loop
^52^	74.5	3.2–7	4	12.5 × 12.5	Not Available	Square coaxial waveguide and vias
^53^	45	22–34.7	2	2.53 × 2.53	FR4	Circular ring
^54^	75.7	1–2.1 and 3.3–4.04	2	5.418 × 5.418 and 2.451 × 2.451	FR4	classical Jerusalem Cross
^55^	182.65	0.31–6.84	2	33.08 × 33.08	PET (Polyethylene terephthalate)	Circles with bending slots
Proposed Design 1	94.3	6.81–18.97	2	11.53 × 11.53	Rogers RO4003C	Cross-dipole and outer ring
Proposed Design 2	85.8	22.28–55.78	2	4.24 × 4.24	Rogers RO4003C	Cross-dipole and outer ring

## Conclusion

A novel wide bandstop FSS resonating at X, Ku and mmWave band is proposed. This miniaturized and wide-bandstop FSS is printed on both sides of a single layer substrate, which is its considerable advantage. The proposed FSS possesses versatile topology and can be easily deployed for lower and higher frequencies. It provides a broad −10 dB bandwidth of 12.16 GHz (from 6.81 to 18.97 GHz), which is equivalent to about 94.5% fractional bandwidth with respect to the center frequency (12.4 GHz) and a −10 dB bandwidth of 33.5 GHz (from 22.28 to 55.78 GHz, equivalent to 85.8% fractional bandwidth with respect to the center frequency of 39 GHz). Also, it exhibits a symmetrical, polarization independent nature. Since it operates at X, Ku and mmWave bands, it can be used for millimeter wave shielding applications and for mutual coupling reduction between antennas. A prototype of the proposed FSS design at X and Ku band has been fabricated and measured. The experimental results are in good agreement with the simulations.

## Data Availability

All data generated or analysed during this study are included in this published article.
